# Dissecting human disease with single-cell omics: application in model systems and in the clinic

**DOI:** 10.1242/dmm.036525

**Published:** 2018-11-05

**Authors:** Paulina M. Strzelecka, Anna M. Ranzoni, Ana Cvejic

**Affiliations:** 1 Department of Haematology, University of Cambridge, Cambridge CB2 0XY, UK; 2 Wellcome Trust Sanger Institute, Wellcome Genome Campus, Hinxton CB10 1SA, UK; 3 Wellcome Trust – Medical Research Council Stem Cell Institute, Cambridge CB2 1QR, UK

**Keywords:** Disease, Model organisms, Single-cell omics

## Abstract

Probing cellular population diversity at single-cell resolution became possible only in recent years. The popularity of single-cell ‘omic’ approaches, which allow researchers to dissect sample heterogeneity and cell-to-cell variation, continues to grow. With continuous technological improvements, single-cell omics are becoming increasingly prevalent and contribute to the discovery of new and rare cell types, and to the deciphering of disease pathogenesis and outcome. Animal models of human diseases have significantly facilitated our understanding of the mechanisms driving pathologies and resulted in the development of more efficient therapies. The application of single-cell omics to animal models improves the precision of the obtained insights, and brings single-cell technology closer to the clinical field. This Review focuses on the use of single-cell omics in cellular and animal models of diseases, as well as in samples from human patients. It also highlights the potential of these approaches to further improve the diagnosis and treatment of various pathologies, and includes a discussion of the advantages and remaining challenges in implementing these technologies into clinical practice.

## Abstract (precis)

**Summary:** This Review summarises the current state of the art of single-cell techniques and their potential applications in deciphering the heterogeneous nature of diseases and tailoring personalised therapies.

## Introduction

Human body tissues are heterogeneous environments composed of numerous types of cells with unique functions and at various stages of differentiation. Until recently, the understanding of disease mechanisms progressed by studying cell populations in bulk. The main limitation of this approach is that it reveals only the average features of the population's constituents and can obscure the cell-to-cell variability present in all tissues. Indeed, an average trait within a population is often not representative of the state of any individual cell (Altschuler and Wu, 2010). Even within populations that are homogeneous in terms of cell surface markers, hidden cell-to-cell variations have direct and significant consequences on the cell function. Recent advances in methodology and cost effectiveness of high-throughput ‘omic’ technologies have enabled their application to the study of single cells, allowing a complete and unbiased analysis of the content of individual cells. The technical aspects of the many single-cell omic approaches are available in several excellent reviews (Clark et al., 2016; Gawad et al., 2016; Mincarelli et al., 2018; Svensson et al., 2018) and are not covered here. The aim of this Review is to summarise the current applications of single-cell omics in model organisms and in humans, and to highlight the potential of these approaches for improving the diagnosis and treatment of diseases.

## Omic technologies with single-cell precision

### Single-cell genomics

Single-cell DNA sequencing can be used to resolve the variation between individual cells at the genomic level. The most common parameters assayed in this type of analysis include the number of single-nucleotide variants (SNVs), which occur at an estimated rate of ∼1500 per human cell (Lodato et al., 2015), and subchromosomal copy-number variants (CNVs), which are thought to develop at least once in 30-70% of cells (Knouse et al., 2016). CNVs can be detected at a low sequencing coverage (<1×), whereas SNV analysis typically requires a higher sequencing depth (15-50×) (Woodworth et al., 2017). Other somatic mutations include the insertion of retroelements (see Glossary, Box 1), the rate of which is estimated to be <1 per cell, and variations in microsatellites (Box 1), which are believed to be the most highly variable regions of the genome (Woodworth et al., 2017). As the DNA content of a single cell is estimated to be 6 pg, which is not sufficient as an input for sequencing, several amplification methods have been established (Gawad et al., 2016; Wang and Song, 2017). While most single-cell genome sequencing studies have profiled up to hundreds of cells, a combinatorial indexing technique has recently enabled the sequencing of more than 15,000 cells, with the coverage level suitable to identify CNVs (Vitak et al., 2017).

**Box 1. Glossary**

**Allelic dropout:** loss of one allele during polymerase chain reaction (PCR) amplification of DNA. It represents a common source of error in genotyping.

**Amyloid beta (Aβ):** a peptide generated from the amyloid precursor protein by γ-secretase cleavage. The accumulation of Aβ in the brain is proposed to be involved in the pathogenesis of Alzheimer's disease.

**Bisulfite genomic sequencing:** a method used for the detection of DNA methylation. It is based on bisulfite treatment, which converts non-methylated cytosines into uracil residues, while leaving methylated cytosines unchanged, thus allowing their distinguishing.

**Cancer stem cells (CSCs):** a rare population of cells found in solid tumours and haematological malignancies able to self-renew and differentiate, and therefore act as a reservoir of cancer cells that can cause a relapse after successful treatment.

**Circulating tumour cells (CTCs):** cells that disseminate from the tumour site and enter the circulation. They are known biomarkers in liquid biopsies and their concentration in patient blood is used as a prognostic parameter.

**gDNA-mRNA sequencing (DR-seq):** a method for the simultaneous detection of DNA and RNA from the same sample. Here, genomic DNA and complementary DNA, generated by reverse transcription from RNA, are amplified together before being separated for further reactions.

**Genome and transcriptome sequencing (G&T sequencing):** a method for the simultaneous detection of DNA and RNA from the same sample. In this method, mRNA and gDNA are physically separated from the cell lysate using beads that capture the polyadenylated portion of mRNA, and amplified separately.

***In situ*** **hybridisation:** a method that uses labelled complementary probes to detect specific DNA or RNA sequences in a tissue.

**Microfluidic device:** an instrument that uses small amounts of fluids in miniaturised channels to perform laboratory tests.

**Microsatellites:** repeated sequences of DNA that represent highly variable regions of the genome.

**Multiplexed error-robust fluorescence *in situ* hybridisation (MERFISH):** a method for the detection and quantification of RNA molecules within the histological context. This technique is based on combinatorial *in situ* hybridisation labelling and sequential imaging.

**Myeloma:** a form of bone marrow cancer arising from plasma cells.

**Narcolepsy:** a neurological sleep disorder associated with the destruction of orexin-producing neurons.

**Quantitative hybridisation chain reaction (qHCR):** a method for the quantification of mRNA expression with subcellular resolution. It is based on DNA probes that hybridise the target and initiate the assembly of fluorescent polymers.

**Retroelements:** mobile elements of eukaryotic genomes, constituting nearly 50% of the human genome, which are able to transpose to other locations of the genome through an RNA intermediate.

**RNAscope:** an *in situ* hybridisation assay that enables the detection of RNA sequences within intact tissues and cells.

**Soluble amyloid precursor protein alpha (sAPPα):** a peptide generated from amyloid precursor protein by the α-secretase cleavage. Generation of sAPPα precludes Aβ generation from the same precursor molecule.

**Spatial transcriptomics:** a technique that enables the examination of the spatial distribution of mRNA from RNA sequencing data in the tissue sections.

**Transposase-accessible chromatin sequencing (ATAC-seq):** a method to study genome-wide chromatin accessibility, using Tn5 transposase to insert sequencing primers into regions of open chromatin.

**Transposome hypersensitivity side sequencing:** a highly sensitive method to characterise chromatin accessibility. In contrast to ATAC-seq, it uses a customised Tn5 transposome system to attach a T7 promoter to the end of every DNA molecule after *in vitro* transposition.

Cancer biology is one of the research areas that greatly benefited from the application of single-cell DNA sequencing. Tumours are mosaic tissues arising from different clones, and single-cell DNA sequencing is a powerful tool for following the progression and expansion of individual clones (Gawad et al., 2016; Navin et al., 2011). In addition, single-cell DNA sequencing allows researchers to study the genetic alterations of rare cell types, such as cancer stem cells (CSCs; Box 1), which are important for tumour relapse and would otherwise be overlooked by traditional, bulk analyses (Liu et al., 2017). With single-cell DNA sequencing, researchers can reconstruct cell lineage trees with high precision by detecting somatic mutations that occur in every DNA replication (Frumkin et al., 2005). Nevertheless, many challenges remain to be solved in the single-cell genomic analysis, including allelic dropouts (Box 1), low and non-uniform coverage of large genomes and false-positive errors, in addition to relatively high costs (Navin, 2014; Sabina and Leamon, 2015; Mincarelli et al., 2018).

### Single-cell epigenomics

Although bulk-level studies have identified key epigenetic signatures correlated with active or inactive transcriptional states, this approach fails to detect intercellular differences that can have functional consequences (Bheda and Schneider, 2014). Identifying epigenetic events at the single-cell level is particularly informative during development, whereby a small number of cells are particularly affected by epigenetic changes (Clark et al., 2016). As transcriptional repression is closely associated with cytosine methylation, the single-cell variant of bisulfite genomic sequencing (Box 1) has been developed, allowing the detection of the methylation status of CpG sites (genomic regions characterised by the presence of a cytosine nucleotide followed by a guanine one) across the genome. The main limitation of this method is poor genome coverage (20-40%) (Smallwood et al., 2014). Single-cell techniques can also assess chromatin accessibility. The combination of multiplex barcoding and transposase-accessible chromatin sequencing (ATAC-seq; Box 1) allows the simultaneous investigation of the chromatin state in 15,000 cells, albeit with low sequencing depth (Cusanovich et al., 2015). Despite the recent advances, single-cell epigenomics is still in its infancy compared with genomics and transcriptomics, and therefore it is not yet widely applied to study the corresponding pathologies (Mincarelli et al., 2018).

### Single-cell transcriptomics

Single-cell RNA sequencing (scRNA-seq) technologies have advanced rapidly in recent years. These techniques rely on the conversion of RNA into complementary DNA, which is then amplified to obtain large enough quantities for sequencing. The first transcriptome-wide profiling of a single cell was reported in 2009 (Tang et al., 2009), followed by the development of many other platforms, summarised in a recent review by Svensson and colleagues (Svensson et al., 2018). In particular, sample multiplexing has enabled the analysis of hundreds of cells with 100,000-4,000,000 reads per cell, while droplet-based and nanowell approaches allow several thousands of cells to be analysed, albeit at a lower coverage, with 20,000-200,000 reads per cell (Mincarelli et al., 2018). Studying the transcriptome of individual cells is a useful tool because it allows an unbiased determination of the cell state, representing a step forward from the use of surface markers, wherein cells that homogeneously express such markers can differ substantially in their transcriptome, state and function (Altschuler and Wu, 2010). Recently, the application of scRNA-seq technologies allowed the revisiting and reconstruction of the traditional haematopoietic lineage tree, and showed that haematopoietic differentiation is a continuous rather than a stepwise process (Notta et al., 2016; Athanasiadis et al., 2017; Velten et al., 2017). Technological advances and a wide adoption of scRNA-seq approaches have shifted the application of this method from descriptive analyses of cell heterogeneity towards the understanding of disease mechanisms. We summarise examples in the fields of immunity, cancer and neurodegenerative disorders below.

### Single-cell proteomics

Proteome analysis at the single-cell level could provide essential information on the state and function of a cell. However, analysing the protein content of a single cell is challenging, mainly due to the lack of methods for protein amplification. In recent years, cytometry approaches based on fluorescence-activated cell sorting (FACS) and single-cell mass spectrometry (termed CyTOF) became available for medium-throughput studies (∼40-50 proteins), but are limited by the availability of the corresponding antibodies (Mincarelli et al., 2018). Furthermore, approaches based on liquid chromatography and tandem mass spectrometry have been successfully used to detect up to 450 proteins in single oocytes. Nevertheless, the analysis of smaller cells, which contain lower amounts of proteins, remains challenging (Virant-Klun et al., 2016).

### Single-cell multiomics

The past few years have seen the rise of the so-called multiomics at the single-cell level, involving the simultaneous analysis of multiple molecular features within the same cell. Such parallel analysis of the genome and transcriptome is appealing because it links the genotype of a cell to its phenotype. Additionally, it represents a powerful tool for the reconstruction of developmental lineage trees, because of the possibility to follow the somatic mutations that accumulate in the genome over time, and at the same time to link those to the phenotypic changes in the cell (Macaulay et al., 2017). Two of the most common methods in single-cell multiomic studies are genomic DNA (gDNA)-messenger RNA (mRNA) sequencing (DR-seq; Box 1) (Dey et al., 2015) and genome and transcriptome sequencing (G&T-seq; Box 1) (Macaulay et al., 2017), which allow the simultaneous interrogation of DNA and RNA from a single cell. Conversely, the concurrent study of the epigenome and transcriptome of a cell gives useful insights into the regulation of gene expression, and can help to elucidate the individual contributions of epigenetic and transcriptional changes to the cell commitment and fate choice. Accordingly, several approaches that combine single-cell bisulfite genome sequencing and RNA sequencing have been developed (Guo et al., 2013; Smallwood et al., 2014). Researchers also attempted to develop tools for the parallel detection of proteome and transcriptome in the same cell, but the single-cell proteomic technologies are not yet sensitive enough to cover large numbers of proteins, and further advances in technology are needed to achieve the necessary wider coverage (Macaulay et al., 2017).

## Single-cell omics applied to disease

### Infections: host-pathogen interactions at the single-cell level

**Fig. 1.**
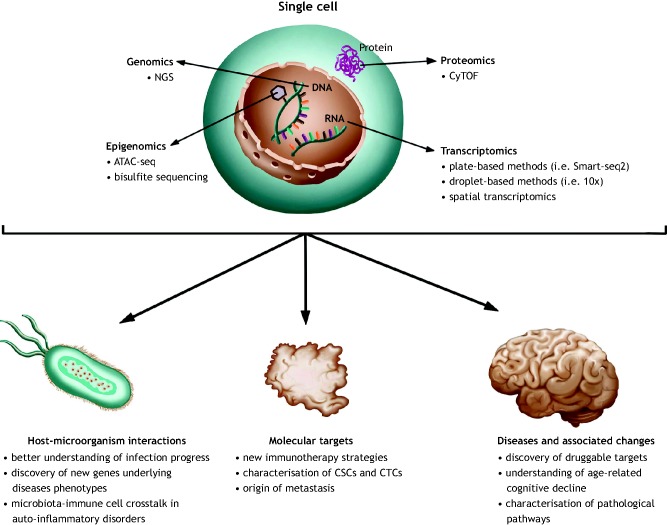
**Application of single-cell omic technologies to study diseases.** Combined information about the transcriptome, genome, proteome and epigenome of a single cell, obtained with constantly evolving technologies, will drive the progress of personalised medicine and generation of improved targeted therapies. ATAC-seq, transposase-accessible chromatin sequencing; CSCs, cancer stem cells; CTCs, circulating tumour cells; CyTOF, cytometry time-of-flight mass spectrometry; NGS, next generation sequencing.

Infectious diseases are one of the leading causes of mortality worldwide (Fauci, 2001). Host-pathogen interactions underlie the disease pathogenesis and determine the outcome of the infection (Fig. 1). These interactions are highly diverse and complex: both pathogens and immune cells display extensive cell-to-cell variability, and both undergo changes in gene expression upon infection (Avraham et al., 2015; Saliba et al., 2016). Therefore, traditional bulk assays are poorly suited to study these interactions, as they neglect important cell-to-cell variation that contributes to the infection outcome. The development of single-cell technologies is providing unprecedented insight into host-pathogen interactions at the single-cell level, allowing the better identification of virulence agents and mechanisms of host defence. Single-cell approaches have already been applied to study viral, bacterial and parasitic infections, as well as the interactions between commensal bacteria and host immune cells (Avraham et al., 2015; Guo et al., 2017; Gury-BenAri et al., 2016; Reid et al., 2018; Saliba et al., 2016; Steuerman et al., 2018).

Several reports have described host-virus interactions at the single-cell level, mostly using fluorescent techniques to monitor virus behaviour in the infected cells (Akpinar et al., 2016; Schulte and Andino, 2014; Warrick et al., 2016). For instance, Guo et al. created a microfluidic device (Box 1) combined with a fluorescent microscope to evaluate the kinetics of poliovirus infection in individual cells (Guo et al., 2017). This single-cell resolution revealed the variation in virus replication kinetics between individual cells. Moreover, they showed that the virus replication potential was host cell dependent, indicating that host cell population heterogeneity influences the outcome of a viral infection. Steuerman at al. utilised scRNA-seq in a mouse model to investigate heterogeneity in the response of lung tissue cells to an influenza infection (Steuerman et al., 2018). The method simultaneously mapped the viral and host transcriptomes in the same cell, and could be applicable to a wide range of viruses with polyadenylated transcriptomes, including both negative-sense single-stranded RNA viruses (influenza, Ebola, measles) and double-stranded DNA viruses (herpes viruses, adenoviruses, pox viruses). The main advantage of implementing single-cell technology in this study was the discovery of novel specific markers that distinguish influenza-infected cells. Overall, virology studies at the single-cell level demonstrate that the description of a population of infected cells based on the behaviour of a single cell allows for better qualitative and quantitative examination of an infection progress. Single-cell omics have great potential in deciphering virus biology and virus-host cell interactions, and are a powerful tool in virology that should be applied more often in the future.

In malaria biology, a number of persistent questions could benefit from a single-cell level investigation. At the Sanger Institute, scRNA-seq is currently used to understand the transcriptional diversity across the full life cycle of the malaria parasites under different disease settings (Reid et al., 2018). The Malaria Cell Atlas (MCA, https://www.sanger.ac.uk/science/tools/mca) has been generated as a part of an initiative aiming to eradicate malaria. This resource provides information about transcriptomes of hundreds of *Plasmodium* isolates. In-depth transcriptional analysis of the parasite at the single-cell level might provide useful information in terms of drug-targeting strategies. Nearly half of the world population is at risk of developing malaria, and individuals can be infected with multiple strains of the parasite. Transcriptional variation in parasites is associated with critical disease phenotypes, including red blood cell invasion and immune evasion (Reid et al., 2018). *Plasmodium* species have a complex life cycle with many different developmental stages. Although the parasite's life-cycle stages have been extensively investigated at the transcriptional level, these studies were until recently conducted using only the bulk-cell-population approach (Otto et al., 2014; López-Barragán et al., 2011; Lasonder et al., 2016). Insight into individual cells may uncover distinct transcriptional signatures responsible for pathogenesis. Recently, Reid et al. used scRNA-seq analysis to delineate transcriptional variation of human and rodent malaria parasites by applying this sequencing method to FACS-sorted, parasite-infected red blood cells (Reid et al., 2018). The researchers optimised the standard Smart-seq2 protocol (Picelli et al., 2013) to improve the percentage of genes mapped to the parasite genome. This enabled not only the identification of different cell types, but also the exploration of the functional variation among individual cells. Moreover, single-cell gene expression analysis performed in this study revealed abrupt changes in *Plasmodium* gene expression during the asexual cycle, in contrast to previously published results of the bulk-level experiments that showed a smooth transition (Bozdech et al., 2003; Hoo et al., 2016). As the asexual replication cycle of *Plasmodium* is linked to pathogenesis, these results are important for improved understanding of the infection progress and for the development of potential therapies. In summary, the application of single-cell transcriptome profiling to study malaria infection allows the discovery of the genes underlying important disease phenotypes.

Animal bodies host a diverse collection of microorganisms that comprise a complex microbiome, which plays an important role in both homeostasis and disease (Tolonen and Xavier, 2017). Bacterial populations consist of a broad spectrum of individual cells, making them the ideal targets to be studied using single-cell omic approaches. Specifically, single-cell genomics hold the potential to decipher complex interactions between immune cells and the microbiota. Although bacteriologists have already been implementing single-cell imaging to observe cell growth and division, the development of single-cell omics enables the detailed profiling of bacterial RNA, DNA, protein and metabolites. Advances in the sequencing of single microbial cells could help uncover the functional roles of the human microbiome members. Insight into the interactions of immune cells with the microbiome might delineate their importance in health, which is especially relevant for the many chronic diseases that are associated with changes in microbiota. Recently, Gury-BenAri et al. applied single-cell technologies to study the effect of a perturbed microbiome on murine intestinal innate lymphoid cells (ILCs) (Gury-BenAri et al., 2016). Using germ-free or antibiotic-treated mice, the authors showed significant changes in the ILC transcriptome caused by a perturbed microbiome, with a global acquisition of genomic elements characteristic of type 3 ILCs that are associated with inflammatory bowel disease. This discovery highlights the importance of ILC-microbiota crosstalk in creating a healthy intestinal microenvironment and preventing auto-inflammatory disorders. In addition to uncovering the pathogenesis of chronic diseases, analysis of individual genomes from microbiota can also greatly benefit other fields, such as epidemiology, by tracing the emergence of pathogenic and drug-resistant strains.

The application of single-cell gene expression analysis can also highlight differences in the responses of individual immune cells to pathogen heterogeneity. Avraham et al. developed an experimental setup that combined scRNA-seq with fluorescent markers in a mouse model to study a *Salmonella* infection (Avraham et al., 2015). Fluorescent labelling allowed the authors to distinguish between uninfected macrophages, infected macrophages containing dead bacteria and infected ones containing live bacteria. Subsequent scRNA-seq analysis of different macrophage populations identified sets of genes related to the infection phenotypes. Similar results were obtained by Saliba et al., who also used scRNA-seq to show how *Salmonella* infection influences the polarisation of murine macrophages (Saliba et al., 2016). The results revealed that macrophages containing non-growing bacteria displayed pro-inflammatory M1 polarisation, whereas those infected with growing bacteria preferentially differentiated into a population of anti-inflammatory M2 macrophages.

In infection models, single-cell omics can provide information on the heterogeneity of host-pathogen interactions (Fig. 1), revealing the dynamics of an infection and the emergence of drug resistance. Dissecting the infection progress with single-cell precision can lead to the discovery of druggable targets at the early stages of infection, which can therefore result in more effective treatments. Moreover, studying the interaction of human microbiota with the immune cells at the single-cell level can provide valuable insight into how cell function is influenced by a microbiome, and how it is perturbed in a pathological state (Tolonen and Xavier, 2017). Therefore, single-cell omic technologies are emerging as a powerful tool in microbiology. However, some challenges remain. For example, the small size of pathogenic microorganisms translates into lower nucleic acid and protein content compared with that of eukaryotic cells, while the high GC content of microbial genomes hinders their sequencing. Therefore, the successful application of single-cell technologies in microbiology requires constant development of experimental protocols tailored to microbial material (Blainey, 2013).

### Cancer: dissecting tumour heterogeneity

The application of single-cell omics could answer several major questions in cancer biology. These include the identification of distinct molecular patterns involved in disease progression and relapse, and the mechanisms of tumour immune evasion (Fig. 1). Intratumour heterogeneity is well documented, and has been linked to clonal evolution, rare cell populations and dynamic cell states (Levitin et al., 2018). The tumour microenvironment consists of a heterogeneous population of cells including malignant, stromal and infiltrating immune cells (Bussard et al., 2016). Therefore, tumour phenotyping at the bulk level does not provide sufficient information on the molecular profile of tumour cells. Indeed, single-cell technologies have been successfully applied in cancer biology to analyse intratumour heterogeneity at multiple molecular levels, including the genome, transcriptome and proteome, opening opportunities for in-depth determination of key molecular tumour properties that could influence the clinical outcome.

With the view of designing optimised strategies for immunotherapy, Lavin et al. recently deciphered the immune signature of lung adenocarcinoma at the single-cell level. A combination of a barcoding method and mass cytometry allowed for simultaneous single-cell analysis of the tumour, the non-involved lung tissue and the peripheral blood. This approach revealed a unique tumour-immune signature and provided a detailed immune cell atlas of the human lung (Lavin et al., 2017). Comparative analysis of the tumour and the normal lung delineated changes associated with tumour progression, emphasising the relevance of such analysis for understanding the tumour microenvironment (Lambrechts et al., 2018). Performing single-cell analysis on multiple tissues not only provided a detailed cellular map of lung adenocarcinoma but also identified tumour-specific changes. Identification of such changes will help to develop immunotherapy strategies tailored to restore a normal immune signature.

Single-cell omic approaches are also a powerful tool to detect and characterise rare populations of tumour cells, such as circulating tumour cells (CTCs; Box 1) or CSCs. Being quiescent, CSCs are resistant to standard chemotherapy and can sustain the long-term growth of cancer (Giustacchini et al., 2017). The genetic diversity of a tumour is linked to the diversity of its stem cell population (Lawson et al., 2015). Lawson et al. applied single-cell transcriptomics to study heterogeneity within a stem cell pool in breast carcinoma (Lawson et al., 2015). They generated the expression signature of normal breast epithelium and used it as a reference to resolve differentiation pathways in metastatic cells obtained from patient-derived mice xenografts. Their study revealed that metastatic cells from low-burden tissues – those with a low number of cancer cells – are distinct from the primary tumour cells. By contrast, cells from high-burden tissues – those containing a high number of cancer cells – were more similar to the primary tumour cells. The single-cell gene expression analysis indicated that metastatic cells from breast cancer have a stem-cell-like transcriptional signature. They can give rise to differentiated progeny once seeded within a distant tissue. The single-cell approach uncovered the diversity of differentiation and gene expression at the metastatic stage, which could potentially aid in the design of drugs that target metastatic disease. Furthermore, Giustacchini et al. looked at the molecular signature of stem cells in chronic myeloid leukaemia (CLM), applying scRNA-seq to analyse the CSCs (Giustacchini et al., 2017). The CSC compartment in CLM is well established, and the persistence of these cells during therapy poses a continuous challenge. CLM-derived CSCs are characterised by the presence of a somatic mutation – a *BCR-ABL* fusion gene. As scRNA-seq lacks the sensitivity to reliably detect somatic mutations, Giustacchini and co-workers modified the standard Smart-seq2 method (Picelli et al., 2013) to combine a highly sensitive mutation detection with transcriptome analysis in the same cell. This new technique allowed them to selectively analyse aberrant CSC gene expression at the time of diagnosis, as well as during treatment-mediated remission. The data showed that treatment-resistant CSCs were transcriptionally different from normal haematopoietic stem cells, highlighting the genes that could be selectively targeted. This study illustrated how single-cell omic analysis can identify a population of therapy-resistant CSCs and provide opportunities for the development of targeted therapies.

The other rare population of cancer cells, CTCs, is also highly suited for single-cell analysis. In-depth analysis of CTCs has important clinical implications, as serial sampling of blood containing CTCs allows the monitoring of cancer progression over time (Lapin et al., 2017; Ramsköld et al., 2012). The work of Miyamoto and colleagues showed that studying the transcriptomes of single CTCs in prostate cancer allows researchers to monitor the acquisition of genetic changes resulting in acquired therapy resistance (Miyamoto et al., 2015). This is a great example of how therapy can benefit from the application of single-cell technologies. Ideally, further development of these techniques in monitoring the CTC pool will contribute to improved understanding and management of drug resistance. It will allow patients to be switched to a different drug before resistance arises. Several other studies also utilised single-cell transcriptomics to examine CTCs. For instance, Ramsköld et al. developed a Smart-seq protocol to characterise melanoma-derived CTCs (Ramsköld et al., 2012), whereas Lapin et al. performed mRNA profiling of CTCs from pancreatic cancer patients using single-cell multiplex reverse transcription quantitative PCR (Lapin et al., 2017). Both studies revealed distinct gene expression signatures within CTC pools, and again confirmed that single-cell data can capture the true diversity of cell populations that bulk-level analyses cannot.

The main cause of mortality among cancer patients is metastasis (Mehlen and Puisieux, 2006). A better understanding of the mechanisms underlying cancer metastasis connected with disseminated tumour cells (DTCs) can lead to more effective treatments. Currently, there are two models explaining the origins of DTCs during tumour evolution: parallel and linear (Klein, 2009). The parallel model suggests that cancer cells disseminate from the primary tumour early, whereas, according to the linear model, DTCs leave the primary tumour site sequentially. Therefore, depending on which model is applied, DTCs should have either profoundly different or similar genomes compared with the primary tumour, respectively. Single-cell genomics offers the possibility to trace the origin of DTCs and to evaluate the proposed cancer progression models. Demeulemeester et al. used this method to study DTCs from breast carcinoma patients (Demeulemeester et al., 2016). The researchers sequenced genomic DNA from single DTCs isolated from bone marrow aspirates. Bulk primary tumours and lymph node samples were subjected to single-nucleotide polymorphism array profiling and whole-genome sequencing. The genomic profiles of single DTCs were correlated with the profile of bulk samples to trace the origin of DTCs. The results showed that mutation profiles of DTCs and of the primary tumour were similar, suggesting that breast cancer cells either acquire the ability to disseminate from the primary site late in their evolution or that continuous seeding and replacement occurs. In other words, early disseminating tumour cells are replaced by late ones as they compete for the bone marrow niche. This study showed that single-cell sequencing is a powerful tool to better understand the origin of metastasis.

Owing to their precision, single-cell omics can also be utilised when choosing the appropriate treatment regimen for cancer patients. Kim and colleagues applied scRNA-seq analysis to optimise the treatment strategy for metastatic renal cell carcinoma patients (Kim et al., 2016). They generated patient-derived mouse xenografts from primary tumours or lung metastases. Single-cell transcriptome analysis revealed that cells from the metastasis xenografts had a different drug sensitivity signature than cells from the primary tumour xenografts. The study showed that molecular targeted therapies could be designed based on prediction signatures obtained from scRNA-seq data. Mitra et al. also applied single-cell transcriptomics to elucidate the drug sensitivity/resistance profile of myeloma (Box 1) (Mitra et al., 2016). The researchers demonstrated that the single-cell gene expression signature can predict the sensitivity or resistance to anticancer drugs. Moreover, they also used a machine-learning approach to select the most relevant genes necessary for predicting the drug response in individual cells. These studies highlight that single-cell omics can facilitate the individualised clinical decision-making process.

Intratumour heterogeneity is the biggest challenge to overcome in the design of targeted cancer therapies. High-throughput single-cell approaches can address this challenge by providing the precision to dissect the heterogeneity within tumour cell populations. Therefore, we expect that these technologies will greatly facilitate the development of targeted cancer therapies in the future. Single-cell transcriptomic profiling can provide information about the expression pattern of potential drug targets, indicating, for instance, whether they are expressed ubiquitously or present only in a specific population of cells. Therefore, single-cell omics will contribute to choosing the right combination of targeted therapies, and enable more accurate patient enrolment in clinical trials (Levitin et al., 2018).

Overall, single-cell omics is a powerful tool that improves our understanding of tumour invasion, metastasis, immune evasion and resistance to therapy (Fig. 1). The application of these technologies to clinical research can improve the early detection and monitoring of cancer, and set guidelines for targeted therapies.

Solid tumours contain multiple microenvironments, and the crosstalk between malignant cells and those microenvironments could influence the response to therapy. The current drawback of single-cell sequencing is the requirement for cellular dissociation, which results in the loss of spatial information. Spatial transcriptomics (Box 1) can address this drawback. It provides information about gene expression data together with visualisation of mRNA distribution within tissue sections. Combining this information allows for novel types of bioinformatics analyses, valuable in research and diagnostics (Stahl et al., 2016). New methods are being developed, such as RNAscope (Box 1) (Wang et al., 2012), multiplexed error-robust fluorescence *in situ* hybridisation (MERFISH; Box 1) (Chen et al., 2015; Moffitt et al., 2016) or quantitative hybridisation chain reaction (qHCR; Box 1) (Trivedi et al., 2018), which allow for *in situ* analysis of biomarkers in the histopathological context.

### Brain disorders: obtaining single-cell details

The human brain consists of ∼100 billion neurons and large populations of non-neuronal cells (Lake et al., 2017). Even in a single brain region, there is significant variation in morphology, connectivity and electrophysical properties between neurons (Lake et al., 2017). Identification of the cell types involved in disease pathogenesis is extremely challenging in a highly heterogeneous tissue like the brain. Therefore, single-cell omic methods have been used to create a detailed map of the brain to trace the origins of cells implicated in the pathogenesis of neuronal diseases (Fig. 1).

Recently, researchers successfully applied CyTOF to better understand the background of narcolepsy (Box 1) (Hartmann et al., 2016). Hartman et al. analysed immune cell populations from the blood of narcolepsy patients or healthy control subjects. Application of CyTOF identified major immune cell populations, determined their activation status, and, most importantly, analysed their chemokine receptor and cytokine expression patterns. Furthermore, this single-cell-resolution study revealed numerous immunological phenotypes associated with narcolepsy and demonstrated the importance of further investigation of lymphocyte populations and their effector mechanisms as therapeutic targets in narcolepsy.

Single-cell omics can also uncover the differences between cell populations in neurodegenerative and neuroinflammatory diseases. For instance, Ajami et al. employed CyTOF to dissect the heterogeneity of myeloid cell populations in mouse models of multiple sclerosis (MS) as an example of neuroinflammatory disease, and Huntington's disease (HD) and amyotrophic lateral sclerosis as examples of neurodegenerative diseases (Ajami et al., 2018). The application of CyTOF enabled an in-depth analysis of cell surface markers, signalling molecules and cytokine profiles of myeloid cells in these diseases. The analysis revealed three different myeloid populations that were characteristic of the central nervous system and absent from peripheral blood. One of the populations was highly enriched in all three pathological conditions compared with healthy controls. Furthermore, analysis of signalling markers showed that myeloid populations in HD lacked the inflammatory signalling signatures present in the MS model. Single-cell insight into the cytokine profiles of all three models revealed that even though myeloid populations across the models were homogenous in terms of cell surface markers, they contained heterogeneous functional subsets, depending on disease aetiology. In the neuroinflammatory disease model, cells were producing multiple cytokines simultaneously, while myeloid cell populations in the neurodegenerative disease models could be distinguished based on the production of a single type of cytokine. This analysis suggested that, although cytokine levels are a general marker of immune response, they should be examined in detail in terms of whether single or multiple cytokines are produced within a cell subpopulation. In summary, Ajami et al. illustrated the power of single-cell proteomics in understanding the heterogeneity of myeloid cells in neuropathologies, and the differences between neuroinflammation and neurodegeneration (Ajami et al., 2018). Single-cell approaches have the potential to further characterise neuroinflammatory and neurodegenerative conditions in the context of druggable targets. As the study was performed in mouse models, the next step would be to extrapolate this approach to human samples.

In another example of successful application of single-cell omics to study human patient samples, Lodato et al. examined somatic SNVs (sSNVs) at the single-neuron level during ageing and neurodegeneration (Lodato et al., 2018). Although previous genomic studies on bulk brain DNA showed the accumulation of somatic mutations with ageing, they could not determine whether mutation occurs specifically in mature neurons. As somatic mutations in postmitotic neurons are cell specific, they can only be comprehensively analysed by comparing the genomes of individual cells. The study demonstrated that sSNVs in neurons accumulate slowly with age. Extending the knowledge of the accumulation of mutations across brain regions will greatly increase our understanding of age-related cognitive decline.

In a recent study, Liao et al. applied single-cell technology to better elucidate the pathogenesis of Alzheimer's disease (AD). The authors developed a method to study the secretome of human induced pluripotent stem cell-derived neural cells (Liao et al., 2016). The method was subsequently applied to measure the levels of amyloid beta (Aβ; Box 1) and soluble amyloid precursor protein alpha (sAPPα; Box 1), which are implicated in AD pathogenesis. Examination of Aβ and sAPPα levels from single cells identified the complex biology of Aβ generation. The study revealed heterogeneity in the secretion levels of Aβ and sAPPα in a homogenous population of cells. Critically, single-cell analysis showed that not only neurons, but also non-neuronal cells, secrete high levels of Aβ. This discovery supported the related recent findings in mouse models of AD (Veeraraghavalu et al., 2014) and shed new light on AD pathogenesis.

Owing to the high level of complexity and heterogeneity of the neurons involved in neurological disorders, developing treatments is truly challenging (Fig. 1). Single-cell omics are promising tools to improve our understanding of the pathological pathways involved in disease initiation and progression that can facilitate the development of effective therapies. So far, single-cell sequencing was successfully applied to several neurological disorders. However, further development of single-cell techniques will most likely result in even broader application within this field. Recently, Lake et al. successfully combined single-nucleus droplet-based sequencing and transposome hypersensitivity side sequencing (Box 1) to study the transcriptome and DNA accessibility profiles of single cells from different regions of the human brain (Lake et al., 2017). This innovative research highlights the power of multiomics in detailed mapping of heterogeneous cell populations to decipher complex tissues like the brain.

## Current limitations and future directions

In recent years, single-cell omics have opened new areas of research and helped to answer biological questions with unprecedented resolution. Despite the rapid development of new technologies, many limitations persist. One of them is the gene expression changes induced by sample processing (van den Brink et al., 2017). Currently, new protocols that minimise dissociation-induced gene expression changes are being developed (Lambrechts et al., 2018). Moreover, generation of single-cell suspensions that are representative of the initial cell population of interest is still challenging, as automated capturing methods usually sample only a fraction of cells (Svensson et al., 2018). In the progress towards the implementation of single-cell multiomics, sensitivity remains the main challenge. Methods that rely on genomic and epigenomic analyses are often hampered by allelic and locus dropouts, and base-level events are regularly not detected in a consistent way across the genome (Macaulay et al., 2017). The majority of scRNA-seq platforms are only able to capture the poly(A) fraction of RNA in a cell, thus missing the variety of microRNAs, long non-coding RNA and histone RNAs that have important regulatory functions. Additionally, only ∼10-20% of RNA is reverse transcribed, which generates technical noise (Kolodziejczyk et al., 2015). New methods have recently been proposed to complement existing single-cell transcriptomic techniques and show promise to fully capture the total RNA content of a cell (Faridani et al., 2016; Hayashi et al., 2018).

The loss of spatial contextualisation in the analysis remains one of the main drawbacks of single-cell omic approaches. Information on the pre-capture localisation of the analysed cells, pivotal for research as well as for diagnostic purposes, is lost when dissociating the tissue into individual cells. Several laboratories have taken up the challenge of developing methods that combine single-cell transcriptomics with spatial characterisation. A promising method, termed ‘spatial transcriptomics’, has been developed based on the superimposition of histological slides on arrays of barcoded reverse transcription primers, although this technique is currently unable to attain single-cell resolution (Stahl et al., 2016). The traditional *in situ* hybridisation (Box 1) approach has recently been modified to reach the single-cell level and simultaneously detect the expression of several genes. Multiplex single-molecule fluorescence *in situ* hybridisation techniques, such as MERFISH, rely on combinatorial labelling and sequential visualisation to analyse the expression of thousands of genes (Chen et al., 2015; Moffitt et al., 2016). An alternative approach, qHCR, achieves accurate quantification of gene expression with subcellular resolution (Trivedi et al., 2018). Further progress of spatially defined single-cell transcriptome analysis will have important consequences on research and diagnostics. Such analysis is especially relevant for cancer biology, whereby the crosstalk between malignant cells and the microenvironment can influence disease progression and response to therapy.

Understanding the mechanisms that underlie diseases at the single-cell level requires constant development of both experimental and computational methods. Analysis of single-cell data is a challenging and multidimensional task. Complexity, noise and unique features of cellular tissues make it difficult to extract meaningful biological information from the single-cell database. Therefore, we can drive valid conclusions about biological heterogeneity based on the single-cell data only if technical variability can be determined, minimised and excluded. This requires constant development of statistical and computational methods. Currently, there is no consensus on the computational methods to be used for the analysis of single-cell omics datasets, and accounting for the technical variations associated with such datasets remains challenging (Stegle et al., 2015).

The next step of the single-cell revolution is to bring it into clinical practice. Single-cell transcriptomics, in particular, has allowed large-scale efforts aimed at the generation of atlases of tissues, organs and entire organisms, including an unbiased catalogue and characterisation of all the individual cells contained in an anatomical compartment. In particular, the Human Cell Atlas currently represents the most ambitious effort, with its main goal to provide a reference map of the healthy and diseased tissues of the human body (Regev et al., 2017; Rozenblatt-Rosen et al., 2017). Once the Human Cell Atlas is generated, it will be possible to compare any diseased tissues with the standard reference, which will provide new tools for diagnostics and personalised medicine approaches.

Overall, rapidly developing single-cell technologies will enable more complex profiling of cells in human health and disease. The combined information about transcriptional state, epigenetic modification and cellular ancestry will drive the progress of personalised medicine and better targeted therapies (Fig. 1). Diagnostic assays will become more powerful once they progress from crude bulk methods to single-cell precision. However, the main challenges that still need to be overcome in order to take single-cell techniques into the diagnostic field include a relatively long analysis time and high costs. Only with the development of new experimental and computational tools will it become possible to implement the single-cell omic technologies into clinical practice.
